# Nanofluidic qPCR unable to detect and serotype Streptococcus pneumoniae in urine samples of hospitalized South African patients with community-acquired pneumonia

**DOI:** 10.1038/s41598-023-48045-0

**Published:** 2023-12-04

**Authors:** Courtney P. Olwagen, Tariro R. Jeche, Lara Van Der Merwe, Marta C. Nunes, Shabir A. Madhi, Vicky L. Baillie

**Affiliations:** 1 South Africa Medical Research Council Vaccines and Infectious Diseases Analytics Research Unit, Faculty of Health Science, University of the Witwatersrand, Johannesburg, South Africa; 2 Department of Science National Research Foundation: Vaccine Preventable Diseases, Faculty of Health Science, University of the Witwatersrand, Johannesburg, South Africa; 3 Centre of Excellence in Respiratory Pathogens, Hospices Civils de Lyon, and Centre International de Recherche en Infectiologie (CIRI), Inserm, Université Claude Bernard Lyon 1, CNRS, UMR5308, U1111 Lyon, France; 4 Wits Infectious Diseases and Oncology Research Institute, Faculty of Health Science, University of the Witwatersrand, Johannesburg, South Africa

**Keywords:** Biological techniques, Bacteria, Infectious diseases

## Abstract

Pneumonia is a major cause of death among adults living with HIV in South Africa, but the etiology of many cases remains unknown. This study evaluated the utility of a nanofluidic qPCR assay to detect and serotype *Streptococcus pneumoniae* in urine samples from patients hospitalized with community-acquired pneumonia (CAP). The nanofluidic qPCR assay was optimized to target 13 pneumococcal serotypes and 4 reference genes. Archived urine samples collected from patients > 15 years of age hospitalized with pneumonia between April 2018 and August 2019 were retrospectively tested using the nanofluidic qPCR assay, BinaxNOW urine antigen test, and standard LytA qPCR. Blood culture was undertaken on a subset of the samples at the discretion of the attending physician. Cohens' Kappa statistics were used to determine the concordance between the methods. Of the 828 adults hospitalized for CAP, urine samples were available in 53% (n = 439). Of those, a random subset of 96 (22%) samples underwent testing. Of the participants included in the final analysis, the mean age was 45.8 years (SD 16.2), 49% (n = 47) were female, 98% (n = 94) were black, and 66% (n = 63) were living with HIV infection. The nanofluidic qPCR method was able to detect PCV13 vaccine strains spiked into urine samples; however, the method failed to detect any pneumococcus in clinical samples. In comparison, 19% of the pneumonia cases were attributed to *S. pneumoniae* using urine antigen testing. Nanofluidic qPCR is unable to detect and serotype *Streptococcus pneumoniae* in urine samples of South Africans hospitalized with CAP.

## Introduction

In Sub-Saharan Africa, pneumonia is a leading cause of infection-related deaths in people ≥ 15 years of age and is responsible for approximately 200,000 deaths annually^1^. The burden of HIV infection in South Africa is high with an estimated 8 million adults living with HIV in 2017^2^. Adults living with HIV have a 25-fold greater risk of developing pneumonia^3^ and have an increased risk of hospitalization (13–19-fold) and death (1.5-fold greater case-fatality ratio)^4^ compared to age-matched HIV-uninfected adults. Despite the high burden of pneumonia, the etiology of many pneumonia cases remains unknown^5,6^ due to limitations in test sensitivity and specificity as well as inappropriate specimen types available for establishing a definite diagnosis^7^. Gold standard culture methods that are commonly used for determining the cause of pneumonia lack sensitivity and results are often negative due to antimicrobial usage prior to sampling^8^. Furthermore, the usefulness of sampling the upper respiratory tract (URT) is limited, as most pathogens identified are common colonizers of the URT thus making it difficult to attribute causality to the etiology of pneumonia^9^. Therefore, sputum, lung aspirates, and pleural fluid are the gold standard samples for determining pneumonia etiology; however, collecting lung aspirates and pleural samples fluid samples is invasive and requires medical expertise and infrastructure^10^. Alternatively, urine samples are a non-invasive alternative, which can be used to identify antigens associated with bacteria causing systemic infections^11^.

The nanofluidic qPCR method has been previously optimized to detect 94 pneumococcal serotypes in respiratory swab samples^12,13^. In this study, we evaluated the utility of a nanofluidic (qPCR) assay (Standard BioTools formerly known as Fluidigm) to detect and serotype *Streptococcus pneumoniae* in urine samples collected from adults hospitalized with pneumonia and compared the findings with BinaxNOW *Streptococcus pneumoniae* antigen test and standard *LytA* qPCR analyses.

## Methods

### Optimization of the nanofluidic qPCR assay

The current analysis was limited to the 13 serotypes targeted by the PCV13 vaccine (1, 3, 4, 5, 6A, 6B, 7F, 9V, 14, 18C, 19A, 19F, and 23F) and 4 pneumococcal reference genes (*LytA, PiaB, Ply,* and *Xisco*), as a proof of concept to evaluate the utility of the method for detecting and serotyping pneumococcus in urine samples. Briefly, standard concentration curves and limits of detection (LOD) for each target serotype were determined by amplifying three replicates of a tenfold serial dilution of purified genomic DNA extracted from reference strains spiked into urine samples with the linear dynamic range being 10^1^–10^7^ copies per standard curve. Target control DNA was harvested from exponential phase cultures (OD600 = 0.1), and the number of colony forming units (CFU)/swabs was estimated from the quantification cycle (Cq) values relative to the standard curve. All assays in the reaction set used had a LOD < 10^3^ CFU/ml except for the reaction set targeting serotype 19F in which the LOD was tenfold more sensitive -i.e., < 10^2^ CFU/ml. Further, the efficiency of the assays ranged from 92 to 105%, and the linear dynamic range (five fold and linearity (R^2^) was < 0.95. Additionally, the accuracy ratio for all reactions was within ± 0.1-fold change while the intra-assay variability (repeatability) and inter-assay variability (reproducibility) for all assays were both within the acceptable range of ± 0.167. All primer/probe pairs were tested with genomic DNA from all PCV13 pneumococcal serotypes, as well as 81 non-vaccine serotype/serogroups, with no cross-reaction detected between the assays.

### Study population

Archived urine samples collected from patients hospitalized at the Chris Hani Baragwanath Academic Hospital and the Bheki Mlangeni Hospital in Soweto, Johannesburg South Africa were retrospectively analyzed. The samples were obtained between April 2018 and August 2019 from hospitalized individuals older than 15 years of age, who were enrolled into a surveillance program for respiratory illness. The study focused on analyzing samples from individuals diagnosed with community acquired pneumoniae (CAP), which was ascertained based on the attending physician diagnosis and recorded using the 10th revision of the International Classification of Diseases (ICD) 10 codes assigned. Study participants presented with at least one of the following systemic symptoms: fever/feverishness defined as a temperature ≥ 37.5 °C, headache, myalgia, or malaise, and at least one of the following respiratory symptoms: cough, sore throat, or shortness of breath. Chest X-rays were done at the discretion of the attending physician; but were unavailable for analysis in this study. The date of commencement of antibiotics administered to the patients was recorded. When possible, urine samples were collected from the participants and stored at the Wits VIDA laboratories at − 80° until assayed.

### Total nucleic acid extraction

Total nucleic acids were extracted automatically from urine samples using the NucliSens easyMAG extraction system (BioMérieux, Marcy l’Etoile, France) following manufacturer's instructions. The extracts were stored at -20ºC until assayed.

### Nanofluidic real-time qPCR testing on the biomark HD system (standard BioTools)

Extracts underwent nanofluidic qPCR testing for the PCV13 pneumococcal vaccine serotypes and the 4 pneumococcal reference genes on the Biomark HD System (Standard BioTools) as described previously^13^; however, the BioTools FLEX Six Gene expression integrated fluidic circuit (IFC) plate was used in place of the 96.96 Dynamic Array integrated fluidic circuit (96.96 IFC). Positive samples were defined as those with a Cq ≤ 37 for a specific serotype and at least three out of the four pneumococcal references genes (Lyt*A*, Pia*B*, *Ply,* and Xisco) being tested.

### BinaxNOW Streptococcus pneumoniae antigen testing

A cotton swab was dipped into thawed urine samples and then inserted into the BinaxNOW *Streptococcus pneumoniae* antigen test card following manufactures instructions. Three drops of Reagent A (citrate/phosphate buffer with sodium lauryl sulfate, tween 20, and sodium azide) was added to the antigen testing card before sealing and the results were read after 15 min. A sample was negative and positive for pneumococcus if one or two coloured lines were present on the device, respectively.

### Real-time qPCR testing using standard ABI 7500 system

The ABI 7500 Real-time PCR system (Applied Biosystems, ABI, Foster City, USA) was used to screen for the *S. pneumoniae* autolysin (*LytA*) pneumococcal gene on the clinical urine samples as previously described (140). Briefly, the qPCR was performed in a 20 µL reaction volume containing 12.5 µL 2xTaqMan gene expression master mix (ABI), 0.313 µL 0.25Mm MGB dye-labelled probe (ABI), mixed with a primer mix, and 5 µL of the extracted total nucleic acid. The cycling conditions included incubation at 50 °C for 2 min followed by an initial activation at 95 °C for 10 min, then 50 cycles consisting of denaturing at 95 °C for 15 s and annealing/extension at 60 °C for 1 min. Positive samples were defined as those with Cq ≤ 37.

### Statistical analysis

Statistical analysis was performed with STATA Version 11.0 (StataCorp, Texas, USA). Student T-test or logistics regression using uni-variate analysis was used to compare the demographic and clinical characteristics between positive and negative pneumococcal cases determined by the BinaxNOW method. The sensitivity and specificity of LytA qPCR and nanofluidic qPCR methods to detect S*. pneumoniae* were determined using McNemar’s test. Kappa statistics was used to compare the concordance between the methods.

### Ethics approval

The study was approved by the Human Research Ethics Committee of the University of Witwatersrand (M200430). Written informed consent was obtained from all study participants at the time of initial enrolment. For minors under the age of 16 years, informed consent was obtained from their parent or legal guardian. All methods were performed in accordance with the relevant guidelines and regulations.

## Results

Of the 828 patients hospitalized for CAP, urine samples were available in 53% (n = 439) for testing. Of those, a random subset of 96 (22%) samples, selected using STATA, underwent nanofluidic qPCR (Standard Biotools), BinaxNOW *Streptococcus pneumoniae* antigen test, and *S. pneumoniae LytA* qPCR testing. There was no difference in the clinical and demographic characteristics of the participants that were and were not selected to be included in this study; Table 1. There were also no differences between participants who did or did not have a urine sample available. Of the participants included in the final analysis, the mean age was 45.8 years (SD 16.2), 49% (n = 47) were female, 98% (n = 94) were black, and 66% (n = 63) were living with HIV infection. Furthermore, of the 96 samples that were included in the final analysis, 31% (n = 30) had blood samples collected for culture; however, *Hemophilus influenzae* was isolated in a single sample only with no organisms detected in the majority (97%, n = 29) of the samples.

**Table 1:** Demographics and clinical characteristics of South African’s > 15 years of age hospitalized with community-acquired pneumonia.

Characteristic	Participants with urine samples tested, N = 96	Participants with urine samples not tested, N = 343	Participants with no urine samples, N = 389
Mean age in years, (SD)	45.8 (16)	48.2 (18)	48.4 (18)
Female, n (%)	47 (49)	192 (56)	216 (56)
Black race, n (%)	94 (98)	269 (78)	376 (97)
Smoking, n (%)	23 (24)	64 (19)	74 (19)
Alcohol consumption, n (%)	23 (24)	67 (20)	73 (19)
Antibiotic use, n (%)	85 (89)	310 (90)	345 (89)
Clinical features			
Fever, n (%)	59 (61)	195 (57)	172 (44)
Cough, n (%)	93 (97)	333 (97)	350 (90)
Shortness of breath, n (%)	85 (89)	302 (88)	298 (77)
Chest pain, n (%)	70 (73)	250 (73)	215 (55)
Malaise, n (%)	90 (94)	326 (95)	255 (66)
Headache, n (%)	45 (47)	145 (42)	140 (36)
Comorbid conditions			
HIV-infected, n (%)	63 (66)	180 (52)	196 (50)
COPD, n (%)	3 (3)	8 (2)	5 (1)
Asthma, n (%)	3 (3)	14 (4)	14 (4)

*SD* Standard deviation, *COPD* Chronic obstructive pulmonary disease, *HIV* Human immunodeficiency virus.

### Urine pneumococcal antigen

Of the 96 participants included in the final analysis, 19% (n = 18/96) tested positive for pneumococcal antigens. While there was no difference in the mean age (43.9 vs. 46.1 years) between the antigen-positive and negative participants, participants with a positive antigen test were less likely to have received antibiotic treatment prior to sample collection (72% vs. 92%; OR 0.21; 95% CI 0.57–0.82; Table 2). Furthermore, there was no difference in the C-reactive proteins, mean white blood cell, platelet, neutrophiles or lymphocyte counts between participants with a positive or negative urine antigen result; however, participants with a positive urine antigen test had lower hemoglobin levels (9.8 g/DL) compared with participants with a negative urine antigens test (11.78 g/DL; p = 0.0078; Table 2).

**Table 2:** Demographics and clinical characteristics of participants with a positive and negative BinaxNOW *Streptococcus pneumonaie* antigen test.

Characteristics	Number of observations	Pneumococcal antigen + (n = 18)	Pneumococcal antigen –(n = 78)	P- value	OR (95%CI)
Mean age in years, (SD)	96	43.9 (18)	46.1 (16)	0.61	–
Female, n (%)	96	9 (50)	38 (49)	0.92	1.05 (0.38–2.93)
Black, n (%)	96	17 (94)	77 (99)	0.25	2.13 (0.51–8.72)
Current smoking, n (%)	96	5 (28)	18 (23)	0.67	1.28(0.40–4.08)
Alcohol, n (%)	96	6 (33)	17 (22)	0.30	1.79 (0.58–5.5)
Antibiotic use, n (%)	96	13 (72)	72 (92)	0.02	0.21 (0.57–0.82)
Days between the collection of urine and the first day of antibiotic use	96			0.16	
0 days, n (%)	39	10 (56)	29 (37)		0.47 (0.17–1.34)
1 day, n (%)	31	3 (17)	28 (36)		
2 days, n (%)	19	5 (28)	14 (18)		
3 days, n (%)	7	0	7 (9)		
Clinical features	96				
Fever, n (%)*	96	13 (72)	46 (59)	0.30	1.81 (0.58–5.58)
Cough, n (%)	96	17 (94)	76 (97)	0.51	0.45 (0.38–5.2)
Shortness of breath, n (%)	96	16 (89)	69 (88)	0.956	1.04 (0.2–5.3)
Chest pain, n (%)	96	15 (83)	55 (71)	0.27	2.09 (0.55–7.9)
Malaise, n (%)	96	17 (94)	73 (94)	0.89	1.16 (0.13–10.6)
Headache, n (%)	96	9 (50)	36 (46)	0.77	1.16 (0.41–3.25)
Comorbid conditions					
HIV positive, n (%)	96	14 (78)	49 (63)	0.12	0.99 (0.97–1.01)
COPD, n (%)	96	0	3 (4)	0.40	–
Asthma, n (%)	96	0	3 (4)	0.40	–
Diabetes mellitus, n (%)	96	0	7 (9)	0.19	–
C-Reactive proteins, **					
Mean CRP, SD	72	186 (36)	140 (16)	0.26	–
< 40mg/l, n (%)	72	2/12 (17)	15/60 (25)	0.53	0.60 (0.12–3.05)
≥ 40mg/l, n (%)	72	10/12 (83)	45/60 (75)		
Full blood count**					
Mean white cell count, SD	88	11.84 (2.08)	11.36 (0.79)	0.81	
Mean hemoglobin blood, SD	88	9.80 (0.8)	11.78 (0.28)	0.0078	–
Mean platelet count, SD	89	287.67 (27.76)	320.94 (20.31)	0.48	–
Mean Neophiles count, SD	13	1.35	94.67 (79)		ND
Mean lymphocyte count, SD	13	1.21	9.03 (6.32)		ND

*OR* Odds ratio, *CI* Confidence interval, *SD* Standard deviation, *COPD* Chronic obstructive pulmonary disease.

p-values from Chi-squared and Student t-test. – Not done: the odds ratio was not calculated for continuous variables or variables with no observations.

*Fever was defined as a temperature of more than 37.5 °C.

**Data unknown for some participants.

ND: Too few observations to calculate p-value.

### *S. pneumoniae LytA* qPCR testing

Of the 96 participants included in the final analysis, 4% (n = 4/96) of the urine samples were *S. pneumoniae* LytA qPCR positive. Notably, the majority of the Cq values for the LytA qPCR positive samples were > 35 (75%, n = 3/4) with a single sample testing positive at 31.67 Cq. Further, using the urine antigen test as the reference method, the concordance with *LytA* qPCR (*Kappa* = 0.03) was poor; with the urine antigen method detecting pneumococcus in 18% (n = 16/96) more of the samples than detected by the LytA qPCR method, while an additional 1% (n = 1/96) of the cases had a reactive LytA qPCR but a negative urine antigen result (Table 3). Furthermore, the LytA qPCR method had a low sensitivity for the overall detection of pneumococcus (sensitivity = 18%, specificity = 75%) compared with the urine antigen test.

**Table 3:** Performance of the BinaxNOW compared with the real-time quantitative polymerase chain reaction for S. pneumoniae autolysin gene (n = 96).

	*S. pneumoniae LytA* qPCR ( +)	*S. pneumoniae LytA* qPCR (−)	Total	p-value	Concordance (*kappa)*	Sensitivity (%)	Specificity (%)
BinaxNOW ( +)	1 (1)	17 (18)	18 (19)	0.0017	0.02	18	75
BinaxNOW (−)	3 (3)	75 (78)	78 (81)				
Total	4 (4)	92 (96)	96 (100)				

*qPCR*- quantitative polymerase chain reaction and LytA-autolysin gene.

Numbers are values (%), p-values of ≤ 0.05 were considered significant.

P-value was determined using the McNemar test.

### Nanofluidic qPCR

Of the 96 samples included in the final analysis, the nanofluidic qPCR method failed to detect pneumococcus in any of the samples, with BinaxNOW, and LytA qPCR detected pneumococcus in 19% (n = 18/96), and 4% (n = 4/96) of the samples.

## Discussion

Our study investigated the utility of a nanofluidic qPCR assay (Standard BioTools) to detect and serotype pneumococcus in urine samples collected from adults hospitalized with clinician-diagnosed pneumonia. While the nanofluidic qPCR method was able detect all PCV13 vaccine serotypes spiked into urine samples, it failed to detect pneumococcus in the clinical samples, whereas the urine antigen test and the *LytA* qPCR method were able to detect *S. pneumoniae* in 19% and 4% of the samples, respectively.

While it is unclear why the nanofluidic qPCR method was unable to detect pneumococcus in any of the urine samples tested, some possible explanations include: (i) The bacterial load of pneumococcus present in the urine may have been too low and therefore below the detection limit of the reaction sets (100–1000 CFU/ml) include in the assay; (ii) The very small input sample volume (2.5ul) might have further compounded the low bacterial load issues and might explain why the *LytA* qPCR method, which uses a higher input volume (10ul), detected pneumococcus (all at very high Cq) in a limited number of the samples; (iii) The high antibiotic usage (89%) in our cohort may have inhibited bacterial growth with no organism detected by blood culture in the majority (97%) of the participants who had a blood sample available for culture; (iv) The concentration of urea might have been high enough in some of the samples to inhibit the qPCR by preventing the primers from annealing especially as dehydration is a common side effect of pneumonia that results in increased levels of urea in the urine^14,15^; (v) The urine samples that we tested in our study were stored − 80 °C for up to three years before testing which could result in the formation of crystals of calcium oxalate, uric acid, and amorphous phosphate that could have degraded the pneumococcal DNA^16^.

The findings from the urine antigen method in our study are similar to a previous study done in the same setting between 2005 and 2007, that reported pneumococcus in 23% of the adults hospitalized with pneumonia^17^, albeit higher compared with studies from Italy^18^ and Thailand^19^, that reported *S. pneumoniae* in 13% and 8–11% of hospitalized adults with CAP, respectively. Furthermore, while the urine antigen test in our study was found to be more sensitive at detecting *S. pneumoniae* compared with the other methods, these results should be interpreted with caution as other studies have found that a proportion of patients with positive blood or sputum cultures have negative antigen tests^20^, antigens may cross-react with closely related streptococci species, and urine antigens can be positive for weeks after the onset of disease^10,21^. Nevertheless, the urine antigen method was able to identify an additional 19% of the pneumococcal cases missed by routine culture and future studies should consider using both methods in conjunction to improve the diagnosis on pneumococcal pneumonia.

The study was limited in that chest X-rays were not available for the participants which are routinely used to confirm a physician’s diagnosis of pneumonia and thus our study could have potentially included non-pneumonia cases. Furthermore, only a subset of the samples included in the final analysis had a blood sample collected for culture and of those none were positive for pneumococcus. Lastly, we did not include an inhibitor control in the qPCR setup which may have controlled for the presence of urea in the samples.

In conclusion, the nanofluidic qPCR method was not useful in identifying *S. pneumoniae* in urine samples, despite being able to detect and serotype *S. pneumoniae* in nasopharyngeal swabs samples^13^. Using the urine antigen test, we were able to report on the overall positivity of *S. pneumoniae* in urine samples collected from hospitalized South African adults; however, we were unable to report on the serotype distribution and other methods such as urine antigen detection (UAD) assay could be an alternative to serotype *S. pneumoniae* in urine^22^*.* Further investigations are needed to develop an alternative method to diagnose pneumococcal pneumonia in adults.

## Data Availability

The datasets used and/or analysed during the current study are available from the corresponding author on reasonable request.
